# Supplementary material for: Accuracy of the interferon-gamma release assay for the diagnosis of tuberculous pleurisy: an updated meta-analysis
Source: PeerJ. 2015 May 21;3:e951. doi: 10.7717/peerj.951 (PMC4451019; doi:10.7717/peerj.951)
Supplement: Supplemental Information 2 — PRISMA 2009 flow diagram. [file peerj-03-951-s002.doc]

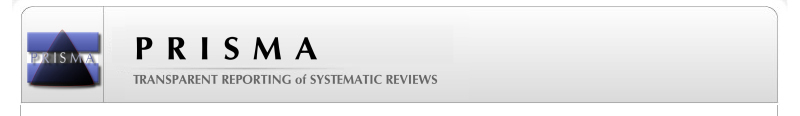
**PRISMA 2009 Flow Diagram**

**Screening**

**Included**

**Eligibility**

**Identification**

Records identified through database searching
(n = 124 )

Additional records identified through other sources
(n = 9 )

Records after duplicates removed
(n =133 )

Records screened
(n = 133 )

Records excluded
(n =83 )

Full-text articles assessed for eligibility
(n = 50 )

Full-text articles excluded, with reasons
(n = 35 )

Studies included in qualitative synthesis
(n = 15 )

Studies included in quantitative synthesis (meta-analysis)
(n =15 )
